# The role of sarcopenia questionnaires in hospitalized patients with chronic heart failure

**DOI:** 10.1007/s40520-020-01561-9

**Published:** 2020-04-28

**Authors:** Wenxue Zhao, Miao Lu, Xiangming Wang, Yan Guo

**Affiliations:** 1 Department of Gerontology, The Second Affiliated Hospital of Nanjing Medical University, 262 Zhongshan North Road, Gulou District, Nanjing, 210003 Jiangsu People’s Republic of China; 2 Department of Gerontology, The First Affiliated Hospital of Nanjing Medical University, 300 Guangzhou Road, Gulou District, Nanjing, 210029 Jiangsu People’s Republic of China

**Keywords:** Sarcopenia, Screening, Sensitivity, Specificity, Chronic heart failure

## Abstract

**Objectives:**

To compare the diagnostic value of the SARC-F, MRSA-7 and MRSA-5 questionnaires in screening for sarcopenia in inpatients with chronic heart failure (CHF).

**Patients:**

A total of 355 CHF patients hospitalized from January 2019 to August 2019 who met the study’s selection criteria were included in the analysis.

**Measurements:**

Handgrip strength and gait speed were measured, and bioelectrical impedance analysis (BIA) was used to estimate appendicular skeletal muscle mass. The sensitivity/specificity of the SARC-F, MRSA-7 and MRSA-5 questionnaires was evaluated.

**Results:**

The diagnostic criteria of the Asia Working Group for Sarcopenia (AWGS) were used as the gold standard for diagnosing sarcopenia. The prevalence of sarcopenia was 55.8% according to the AWGS diagnostic criteria, 31.0% according to the SARC-F, 73.0% according to the MRSA-7, and 71.3% according to the MRSA-5. Using the AWGS criteria as the gold standard, the SARC-F had a sensitivity of 52.5% and a specificity of 96.2% in the whole study population, the MRSA-7 had a sensitivity of 92.4% and a specificity of 51.6%, and the MRSA-5 had a sensitivity of 93.9% and a specificity of 57.3%. The areas under the ROC curves for the SARC-F, MRSA-7 and MRSA-5 were 0.78, 0.74 and 0.77, respectively.

**Conclusions:**

The MSRA-7 and MSRA-5 may serve as novel screening tools for sarcopenia in hospitalized patients with CHF. The SARC-F, a classic screening tool, is also suitable for this population. The MSRA-7 and MSRA-5 have better sensitivity, whereas the SARC-F has better specificity.

**Electronic supplementary material:**

The online version of this article (10.1007/s40520-020-01561-9) contains supplementary material, which is available to authorized users.

## Introduction

Heart failure (HF) is a common chronic disease that is the terminal stage of all kinds of heart disease, and the incidence and prevalence of HF are increasing year by year. In recent years, the peripheral consequences of HF have received more attention [1]. When HF occurs, myocardial contractility decreases, peripheral skeletal muscle blood perfusion is insufficient, and skeletal muscle mass and muscle function decline, leading to sarcopenia. Sarcopenia is a syndrome associated with ageing characterized by a progressive and overall loss of skeletal muscle and strength, which may lead to disability, reduced quality of life and even death. Patients with HF combined with sarcopenia often have weakened muscle strength and decreased exercise ability and are prone to develop weakness and cardiogenic cachexia, increasing the rate of rehospitalization and mortality [2, 3]. Research on sarcopenia in China started late, and research on chronic heart failure (CHF) combined with sarcopenia is limited. Early screening and active intervention are necessary to improve the prognosis of patients with HF complicated with sarcopenia.

According to the gold standard for diagnosing sarcopenia, the diagnosis of sarcopenia relies on large pieces of medical equipment, which is expensive and uncommon. Therefore, testing for sarcopenia cannot be widely promoted, and a simple method for performing a preliminary screening for sarcopenia is needed in clinical practice. Simple methods for screening for sarcopenia include body function assessments, muscle function assessment scales and tools, and questionnaire surveys. Because of the physical limitations of CHF patients, many patients cannot complete body function assessments. The scoring criteria for muscle function assessment tools in the scale category are complex, and there are many scoring principles that are susceptible to the influence of the tester’s motivation. The SARC-F (Supplementary Table S1), MRSA-7 and MRSA-5 (Supplementary Table S2) questionnaires are easy to understand and are not restricted by the health status of the patients, so they are suitable for wide application. However, the accuracy of these three questionnaires has only been verified in healthy people living in the community, and few studies have been conducted in patients with CHF. The purpose of this study was to clarify the accuracy of the three sarcopenia questionnaires in screening for sarcopenia in hospitalized patients with CHF.

## Methods

### Study design and population

A total of 355 consecutive CHF patients hospitalized at the Second Affiliated Hospital of Nanjing Medical University from January 2019 to August 2019, including 187 maintenance haemodialysis (HD) patients, were included. The inclusion criteria were as follows: (1) meeting the 2018 Chinese guidelines for the diagnosis and treatment of HF [4]; presence of typical HF symptoms, exhibiting typical signs of HF, having left ventricular ejection fraction (LVEF) < 40% or LVEF normal or only slightly reduced but associated with structural heart disease and/or diastolic cardiac dysfunction, or having NT-proBNP > 400 pg/ml or BNP > 150 pg/ml; and (2) age ≥ 60 years old. The exclusion criteria were as follows: (1) inability to communicate with researchers or obtain informed consent; (2) major diseases, such as tumours, severe weakness, and survival expectancy of less than 6 months; (3) severe infectious diseases and systemic immune diseases (including systemic lupus erythematosus, connective tissue disease, multiple myeloma, amyloidosis, rheumatism, rheumatoid disease, etc.); (4) acute coronary syndrome and acute HF; (5) chronic pulmonary diseases and liver insufficiency; (6) pregnancy; (7) skin damage at the contact place of the BIA electrode; (8) motor impairment and cognitive impairment; and (9) HF combined with neuromuscular diseases that affect muscle strength measurement or require the long-term use of drugs that affect body weight, such as glucocorticoids.

### Measurement of handgrip strength, gait speed, and skeletal muscle mass

After the patient’s condition was stable and there was no oedema throughout the body, the following measurements were conducted by the researchers before discharge. For the handgrip strength measurement, the patient was seated, and the feet were placed naturally on the ground. The hip was bent 90°, with the upper arm and chest flat, the forearm in a neutral position, the wrist extended 0°–30°, and the feet maintaining a 0°–15° deviation. A mechanical grip dynamometer (xiangshan) was used to measure the strength of the dominant hand twice, and the larger value was recorded. For the gait speed measurement, patients were instructed to walk at a natural speed for 4 m indoors, and the time spent was recorded. The measurement was repeated twice, and the faster value was recorded. The appendicular skeletal muscle mass (ASM) was measured using a bioimpedance analysis (BIA) device (InBody S10, Biospace, Korea). Repeated BIA measurements were performed on all patients. Then, the average of the two measurements was used as the ASM value. The skeletal muscle mass index (SMI) was then obtained by dividing the ASM by height squared. To ensure the same general conditions (clothing, fasting, etc.) during the measurement, two measurements of the same patient were completed within 30 min, and both measurements were conducted by the same researcher. All these tests were performed by a trained researcher.

### Assessment of sarcopenia using different criteria

The diagnostic criteria of AWGS were used as the gold standard for diagnosing sarcopenia (Supplementary Table S3). Next, the SARC-F, MRSA-7, and MRSA-5 were used to estimate sarcopenia. A total score of SARC-F ≥ 4 indicated sarcopenia [5]. A total score of MRSA-7 ≤ 30 and a total score of MRSA-5 ≤ 45 indicated sacopenia [6].

### Covariates

Trained nurses collected the following covariates via face-to-face interviews: age, sex, and medical history of the following chronic diseases: hypertension, diabetes, coronary heart disease and stroke. Additionally, body weight and height were measured using a stadiometer and a digital floor scale to the nearest 0.1 kg and 0.1 cm, respectively.

### Statistical analyses

All statistical analyses were performed with SPSS 20.0 (IBM Corp, Armonk, NY). All statistical tests were two-sided. A *p* value of < 0.001 indicated significance. We also used the ROC curve to compare the overall diagnostic accuracy of the SARC-F, MRSA-7, and MRSA-5. We calculated the area under the ROC curve (AUC).

## Results

### Prevalence of sarcopenia

The prevalence of sarcopenia was 55.8% according to the AWGS diagnostic criteria, 31.0% according to the SARC-F, 73.0% according to the MRSA-7, and 71.3% according to the MRSA-5.

### Patient clinical data

Characteristics of the patients are presented in Table 1. The mean scores of the SARC-F, MSRA-7, and MSRA-5 were all significantly different between the sarcopenic and nonsarcopenic groups (*p* ≤ 0.001) in the whole sample and in males and females separately (Table 1).

**Table 1:** Characteristics of the study population

Characteristics	Total (355)			Men (207)			Women (148)		
	Nonsarcopenia	Sarcopenia	*p*	Nonsarcopenia	Sarcopenia	*p*	Nonsarcopenia	Sarcopenia	*p*
	(*n* = 157)	(*n* = 198)		(*n* = 102)	(*n* = 105)		(*n* = 55)	(*n* = 93)	
Age (years)	67.76 (7.08)	73.58 (10.21)	0.000	67.20 (5.65)	72.92 (10.45)	0.000	68.82 (9.14)	74.32 (9.94)	0.001
Height (m)	1.66 (0.08)	1.63 (0.09)	0.000	1.71 (0.06)	1.69 (0.06)	0.026	1.58 (0.42)	1.55 (0.07)	0.032
Weight (kg)	68.11 ((13.91)	59.72 (11.39)	0.000	72.38 (14.48)	65.38 (11.42)	0.000	60.18 (8.23)	53.32 (7.23)	0.000
HGB (g/L)	117.98 (21.67)	117.60 (24.09)	0.875	123.38 (21.89)	124.48 (28.05)	0.755	107.98 (17.42)	109.84 (15.43)	0.501
TP (g/L)	72.2 (6.61)	68.71 (6.53)	0.000	70.7 8(5.97)	69.18 (6.28)	0.063	74.83 (6.97)	68.17 (6.79)	0.000
ALB (g/L)	41.74 (4.09)	40.91 (4.94)	0.093	42.49 (3.69)	41.67 (4.75)	0.167	40.35 (4.46)	40.06 (5.04)	0.728
Scr (umol/L)	633.26 (441.56)	469.07 (451.48)	0.001	678.73 (470.62)	476.03 (442.72)	0.002	548.93 (371.27)	461.21 (463.45)	0.234
TCH (mmol/L)	3.71 (0.76)	3.49 (0.89)	0.013	3.56 (0.69)	3.26 (0.89)	0.007	3.99 (0.82)	3.74 (0.83)	0.080
TRI (mmol/L)	1.79 (0.94)	1.41 (0.72)	0.000	1.86 (1.10)	1.18 (0.56)	0.000	1.64 (0.51)	1.67 (0.78)	0.799
HDL (mmol/L)	1.03 (0.39)	1.13 (0.30)	0.007	0.9 5(0.30)	1.14 (0.35)	0.000	1.19 (0.47)	1.13 (0.24)	0.274
LDL (mmol/L)	2.2 (0.72)	2.06 (0.80)	0.033	2.15 (0.73)	1.90 (0.79)	0.020	2.38 (0.68)	2.23 (0.78)	0.232
NT-ProBNP (pg/mL)	7378.67 (23,798.95)	11,200.76 (51,981.61)	0.394	4075.54 (5464.19)	16,919.74 (70,722.75)	0.069	4743.85 (7239.79)	13,504.48 (39,007.22)	0.037
Chronic disease									
Hypertension	119 (33.5)	163 (45.9)	0.147	76 (36.7)	88 (42.5)	0.123	43 (29.1)	75 (50.7)	0.833
Diabetes	29 (8.2)	33 (9.3)	0.675	20 (9.7)	6 (2.9)	0.003	9 (6.1)	27 (18.2)	0.112
Stroke	23 (6.5)	92 (25.9)	0.000	14 (6.8)	44 (21.3)	0.000	9 (6.1)	48 (32.4)	0.000
Coronary heart disease	21 (5.9)	52 (14.6)	0.003	18 (8.7)	28 (13.5)	0.134	3 (2.0)	24 (16.2)	0.002
SARC-F score	1.89 (1.22)	4.32 (2.61)	0.000	1.63 (1.35)	4.07 (2.73)	0.000	2.38 (0.73)	4.61 (2.46)	0.000
MRSA-7 score	29.04 (7.79)	22.35 (7.40)	0.000	29.46 (7.80)	23.29 (6.53)	0.000	28.27 (7.77)	21.29 (8.17)	0.000
MRSA-5 score	45.32 (12.98)	33.36 (10.76)	0.000	46.23 (14.04)	34.19 (11.01)	0.000	43.64 (10.65)	32.42 (10.44)	0.000
SARC-F classification									
Nonsarcopenia	151 (42.5)	94 (26.5)	0.000	99 (47.8)	52 (25.1)	0.000	52 (35.1)	42 (28.4)	0.000
Sarcopenia	6 (1.7)	104 (29.3)		3 (1.4)	53 (25.6)		3 (2.0)	51 (34.5)	
MRSA-7 score classification									
Nonsarcopenia	81 (22.8)	15 (4.2)	0.000	49 (23.7)	6 (2.9)	0.000	32 (21.6)	9 (6.1)	0.000
Sarcopenia	76 (21.4)	183 (51.5)		53 (25.6)	99 (47.8)		23 (15.5)	84 (56.8)	
MRSA-5 score classification									
Nonsarcopenia	90 (25.4)	12 (3.4)	0.000	58 (28.0)	6 (2.9)	0.000	32 (21.6)	6 (4.1)	0.000
Sarcopenia	67 (18.9)	186 (52.4)		44 (21.3)	99 (47.8)		23 (15.5)	87 (58.8)	

Mean (SD), *n* (percentage)

*HGB *hemoglobin, *TP *total protein, *ALB *albumin, *Scr *serum creatinine, *TCH *total cholesterol, *TRI *triglyceride, *HDL *high density lipoprotein cholesterol, *DL *low density lipoprotein cholesterol, *NT-ProBNP N*-terminal pro-brain natriuretic peptide

### Sensitivity and specificity

**Fig. 1:**
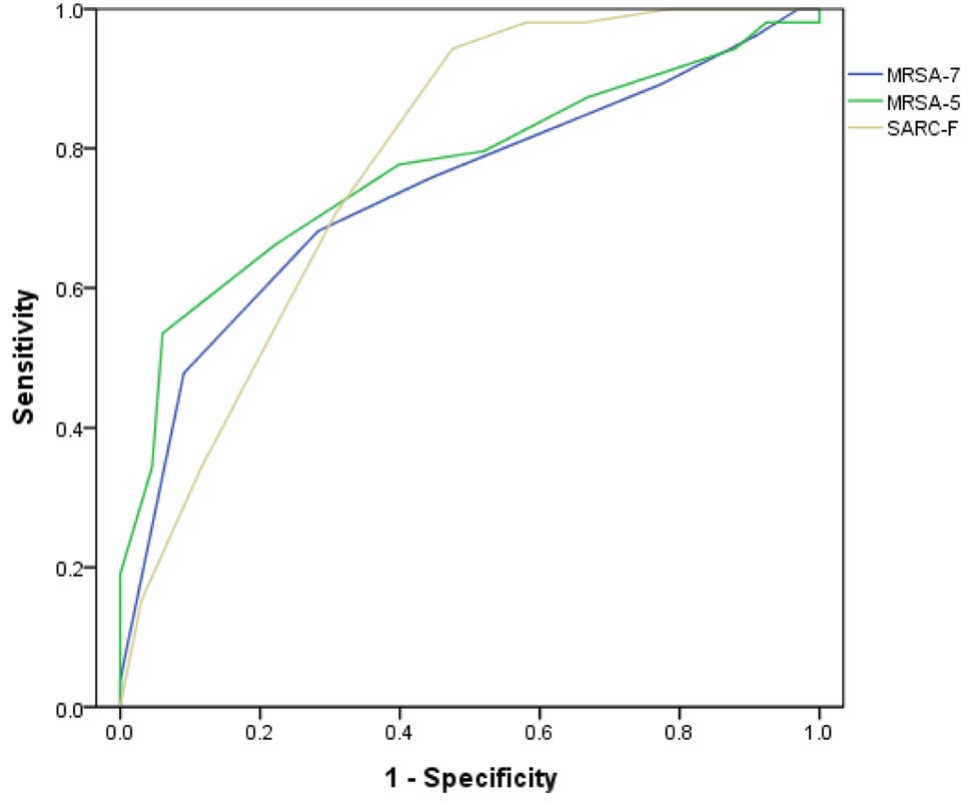
The ROC curves of the SARC-F, MRSA-7, and MRSA-5 against the AWGS in the whole study population

In the entire population, compared with the gold standard of the AWGS, the SARC-F had a sensitivity of 52.5% and a specificity of 96.2%; the MSRA-7 had a sensitivity of 92.4% and a specificity of 51.6%; and the MSRA-5 had a sensitivity of 93.9% and a specificity of 57.3% (Table 2). AUCs of the three scales were very similar (Fig. 1).

**Table 2:** Sensitivity/specificity analyses and ROC models for the SARC-F, MRSA-7 and MRSA-5 validation against the diagnostic criteria of AWGS in the whole study population

		Sensitivity (%)	Specificity (%)	+ LR	−LR	AUC
AWGS	SARC-F	52.5 (45.3–59.6)	96.2 (91.5–98.4)	13.74 (6.20–30.46)	0.49 (0.43–0.57)	0.78 (0.73–0.83)
	MRSA-7	92.4 (87.6–95.5)	51.6 (43.5–59.6)	1.91 (1.62–2.25)	0.15 (0.09–0.24)	0.74 (0.68–0.79)
	MRSA-5	93.9 (89.4–96.7)	57.3 (49.2–65.1)	2.20 (1.83–2.65)	0.11 (0.06–0.18)	0.77 (0.72–0.82)

Table 2 I still miss predictive values that are very important, because they inform about the probability of having sarcopenia when you have a positive (or negative for the negative predictive value) result in the scales.

## Discussion

HF patients often present with fatigue, dyspnoea, and reduced physical strength. These symptoms are not only associated with impaired cardiac pumping, abnormal haemodynamics, and failure of cardiac cells, but also with reduced skeletal muscle function and skeletal muscle cell changes. In recent years, the pathophysiological mechanism of skeletal muscle lesions involved in HF has attracted much attention. Skeletal muscle disease is one of the symptoms of CHF, and its mechanism is via changes in skeletal muscle function, structure, blood flow, metabolism and inflammatory reactions. HF in patients with skeletal muscle lesions includes declining muscle mass and muscle function. Muscle mass decreases mainly because of increased skeletal muscle protein decomposition and reduced synthesis, the transformation of skeletal muscle fibres from type I muscle fibres to type II fast muscle fibres, reduction of capillary distribution density that reduces blood supply, and mitochondrial damage resulting in skeletal muscle loss. In HF patients with sarcopenia, peripheral vascular dysfunction and abnormal skeletal muscle mass and muscle strength lead to decreased exercise tolerance, which is an essential pathophysiological basis for frailty in patients with advanced HF. Patients with HF are prone to frailty, which increases the risk of HF. The frailty-related increase of mortality, disability, and hospitalization is more evident in patients with CHF than those without CHF [7]. Many of the adverse outcomes of frailty may be mediated by sarcopenia, which may be considered the biological substrate for the development of physical frailty and related negative health outcomes [8]. Sarcopenia, frailty, and HF interact to form a vicious cycle [9].

Patients with CHF generally need chest X-ray or chest CT examinations due to the illness. If we measure skeletal muscle mass by CT and so on, not only is the financial burden of the patients increased but the patient’s radioactive ray exposure time is also increased. Most patients with CHF are admitted to the hospital with oedema of the lower limbs and symptoms of chest tightness and asthma, which limit the measurement of skeletal muscle mass by BIA instruments, walking speed and grip strength. The SARC-F, MSRA-7 and MSRA-5 are brief and based on self-reported information. Therefore, they can easily be performed during face-to-face interviews, as self-reported questionnaires, over the telephone, or even through the Internet. The SARC-F has been translated into Korean [10], Chinese [11], Japanese [12, 13], and Spanish [14]. The validity of the SARC-F for screening sarcopenia has also been validated in different ethnic populations. The SARC-F, the early screening tool for sarcopenia, has been widely used in sarcopenia research [15, 16], but few studies involving the SARC-F have been conducted in patients with HF combined with sarcopenia. The MSRA-7 and MSRA-5 are novel screening tools that have not been widely validated in clinical practice. Rossi et al. [6] reported that as pre-screening tool for sarcopenia in the community-dwelling elderly subjects, the sensitivity of the MSRA-7 is 80.4%, and the specificity is 50.5%, while the sensitivity of the MSRA-5 is 80.4% and the specificity is 60.4%. Ming Yang et al. in elderly individuals living in Chinese communities, have found that the sensitivity of the MSRA-7 is 86.9%, and the specificity is 39.6%, while the sensitivity of the MSRA-5 is 90.2%, and the specificity is 70.6% [17]. Our study is the first to validate the accuracy of the SARC-F, MSRA-7 and MSRA-5 in screening for sarcopenia in hospitalized patients with CHF.

The measurement of skeletal muscle mass in our study used the BIA method. In the European Working Group on Sarcopenia in Older People (EWGSOP) [18], the Asia Working Group for Sarcopenia (AWGS) [19], the International Working Group on Sarcopenia (IWGS) [20], and the Foundation for the National Institutes of Health (FNIH) [21] Sarcopenia Project consensus for sarcopenia diagnosis, only the AWGS and EWGSOP recommend the use of BIA to measure skeletal muscle mass. Considering that all of the people we included were Asian, we used the AWGS consensus on sarcopenia as the gold standard. According to the results of Fulster et al.’ s study, the prevalence rate of sarcopenia in patients with mixed heart failure with an average age of 67 was 19.5% [22]. Bekfani et al.[23] indicated that 19.7% of patients with ejection fraction-preserved heart failure had sarcopenia. Kamiya et al. [24] reported that the prevalence of sarcopenia in elderly patients with heart failure was 35.2%. In our study, the prevalence of sarcopenia was 55.8% according to the AWGS diagnostic criteria, 31.0% according to the SARC-F, 73.0% according to the MRSA-7, and 71.3% according to the MRSA-5. Our study showed that the prevalence of sarcopenia in patients with CHF was significantly higher than that in previous studies. The reasons were as follows: ① the enrolled population was HF patients who needed to be hospitalized, and their condition was more serious than that of stable HF patients. ② Our study included 187 patients undergoing long-term haemodialysis (HD), and HF patients undergoing HD perform fewer activities and have more hospitalizations than other patients. ③ Most Chinese individuals do not eat dairy products every day, and some do not even eat dairy products at all. ④ Compared with other previous study populations, our included population needed to restrict liquid intake more strictly, and their dairy intake was lower. These multiple factors led to a significant increase in the prevalence of sarcopenia in our study population.

Compared with the AWGS diagnostic criteria, our study showed that SARC-F screening for sarcopenia in the enrolled population had a low sensitivity (52.5%) and a high specificity (96.2%), while the MRSA-7 and MRSA-5 had a high sensitivity (92.4% for the MSRA-7 and 93.9% for the MSRA-5) and a low specificity (51.6% for the MSRA-7 and 57.3% for the MSRA-5). In our study, the AUCs of the SARC-F, MSRA-7 and MSRA-5 were 0.78, 0.74 and 0.77, respectively, which indicated that the SARC-F, MSRA-7 and MSRA-5 had moderate diagnostic accuracy. In our study, the SARC-F had better specificity, whereas the MRSA-7 and MSRA-5 had higher sensitivity. Therefore, clinicians and researchers should choose the optimal tool according to their specific purpose.

Our study has some limitations. First, we applied BIA instead of the “gold standard” methods (CT, MRI, or DXA) to estimate skeletal muscle mass. Because BIA is a practical and inexpensive method for patients, both the AWGS and EWGSOP recommend using it as an alternative for estimating muscle mass. Second, a small sample size may affect the precision of the accuracy measures obtained. Third, we only compared the SARC-F, MSRA-7, and MSRA-5 questionnaires with commonly used diagnostic criteria in a cross-sectional study. More importantly, the predictive validity of the SARC-F, MSRA-7 and MSRA-5 needs to be tested in longitudinal studies in the future.

## Conclusion

The MSRA-7 and MSRA-5 may serve as novel screening tools for sarcopenia in hospitalized patients with CHF. The SARC-F, a classic screening tool, is also suitable for this population. The MSRA-7, MSRA-5, and SARC-F demonstrated similar overall diagnostic accuracy in our study population. The MSRA-7 and MSRA-5 have better sensitivity, whereas the SARC-F has better specificity. These validations of these tools need to be confirmed in longitudinal studies in the future.

## Electronic supplementary material

Below is the link to the electronic supplementary material.

Supplementary file1 (DOCX 16 kb)
