# Supplementary material for: The role of sarcopenia questionnaires in hospitalized patients with chronic heart failure
Source: Aging Clin Exp Res. 2020 Apr 28;33(2):339–44. doi: 10.1007/s40520-020-01561-9 (PMC7914185; doi:10.1007/s40520-020-01561-9)
Supplement: Supplementary file 1 — Supplementary file1 (DOCX 16 kb) [file 40520_2020_1561_MOESM1_ESM.docx]

**Supplementary Table S1. The SARC-F scale.**

| **Components** | **Questions** | **Score** |
| --- | --- | --- |
| Strength | How much difficulty do you have in lifting and carrying 10 pounds? | None=0  Some =1  A lot or unable = 2 |
| Assistance in walking | How much difficulty do you have walking across a room? | None=0 Some =1  A lot, use aids, or unable = 2 |
| Rise from a chair | How much difficulty do you have transferring from a chair or bed? | None=0 Some =1  A lot or unable without help = 2 |
| Climb stairs | How much difficulty do you have climbing a flight of 10 stairs? | None=0 Some =1  A lot or unable = 2 |
| Falls | How many times have you fallen in the past year? | None=0  1-3 falls =1  4 or more falls = 2 |

**Supplementary Table S2. The MSRA-7 and MSRA-5 scales.**

| **Questions** | **MSRA-7 score** | **MSRA-5 score** |
| --- | --- | --- |
| How old are you？ |  |  |
| ≥ 70 years | 0 | 0 |
| < 70 years | 5 | 5 |
| Were you hospitalized in the last year? |  |  |
| Yes, and ≥2 times | 0 | 0 |
| Yes, but only one hospitalization | 5 | 10 |
| No | 10 | 15 |
| What is your regular activity level? |  |  |
| I can walk <1000 meters | 0 | 0 |
| I can walk ≥1000 meters | 5 | 15 |
| Do you eat three meals every day regularly? |  |  |
| No, up to twice per week I skip a meal (e.g., I skip breakfast or I have only tea or soup  for dinner.) | 0 | 0 |
| Yes | 5 | 15 |
| Do you consume any of the following foods? |  |  |
| Milk or dairy products (e.g., yogurt), but not every day. | 0 | - |
| Milk or dairy products (e.g., yogurt), at least once per day. | 5 | - |
| Do you consume any of the following foods? |  |  |
| Poultry, meat, fish, eggs, legumes, ragout or ham, but not every day | 0 | - |
| Poultry, meat, fish, eggs, legumes, ragout or ham, at least once per day | 5 | - |
| Did you lose weight in the last year? |  |  |
| Yes, and >2kg | 0 | 0 |
| No, or Yes, but ≤2kg | 5 | 10 |

**Supplementary Table S3.The Diagnostic Criteria for Sarcopenia.**

| ① Low muscle mass ② Low HS (kg) ③ Low GS (m/s) Diagnostic criteria | | |
| --- | --- | --- |
| AWGS | SMI<7.0 kg/m^2^ for men; <26 for men; | <0.8 for both sexes ①+② or ①+③ |
|  | SMI<5.7 kg/m^2^ for women <18 for women |  |

AWGS=Asia Working Group for Sarcopenia, SMI=skeletal muscle mass index, HS=handgrip strength, GS=gait speed
